# Performance of Reinforced Concrete Beams Strengthened by Bidirectional Carbon-Fiber-Reinforced Polymers Based on Numerical Models

**DOI:** 10.3390/polym15041012

**Published:** 2023-02-17

**Authors:** Jae Sang Moon, Da Young Kim, Myeong Seop Ko, Changhyuk Kim

**Affiliations:** 1 Structural Department, Yooshin Engineering Corporation, Seoul 06252, Republic of Korea; 2 Department of Architectural Engineering, Inha University, Incheon 22212, Republic of Korea

**Keywords:** CFRP strengthening, bidirectional layout, finite element method, shear strength

## Abstract

The use of carbon-fiber-reinforced polymers (CFRPs) for the repair and rehabilitation of reinforced concrete (RC) structures has been receiving a lot of attention. Specifically, the shear strengthening of RC members based on CFRP materials has been treated as an effective and efficient strengthening method. Previous research projects focused on the shear strengthening of RC members with unidirectional CFRP strips. Although the effectiveness of a bidirectional CFRP layout compared to a unidirectional CFRP layout was discussed in several studies, these studies only investigated the issue based on experiments. Morever, the parameters of the bidirectional CFRP layout were not clearly defined. This study investigates the performance of RC beams strengthened by bidirectional CFRP based on numerical models. A numerical model based on finite element analysis is designed. Using the numerical model, the parameters of the horizontal CFRP strips, such as the layouts of horizontal CFRP strips and the number of horizontal CFRP strips, are studied. The results show that the effect of horizontal CFRP strips is maximized if the strips are distributed along the depth. In contrast, the number of horizontal CFRP strips does not significantly affect the shear strength of RC members.

## 1. Introduction

Although the reinforced concrete structure has been an effective structural system in civil and architectural engineering for a century, the effects of loading and extreme environmental conditions such as earthquakes, typhoons, and floods cause the strength deterioration of the structure. Furthermore, the increase in aged structures (more than 30 years old) has led to the increasing need for structural rehabilitation methods for the structures. To regain the structural performance of RC structures cost effectively, the retrofitting/strengthening of the aging reinforced concrete structures is a must. Various techniques, including section enlargement [1], external prestressing [2], steel plate bonding [3,4,5], and bonding of materials such as fiber-reinforced polymers [6,7], have been applied to improve the performance of aged RC structures. Recently, the use of carbon-fiber-reinforced polymer (CFRP) materials has been widely applied due to their high workability, low cost, and high strength-to-weight ratio. The high corrosion resistance and low thermal conductivity of the CFRP also help engineers to choose the material for the maintenance of RC structures.

Recent studies have focused on understanding the parameters and conditions relating to the use of CFRP in RC structures. Since CFRP-based strengthening is known as an efficient method of shear strengthening [8], many researchers have studied the shear performance of RC structures with CFRP. The contribution of the CFRP materials to the shear strength of RC beams with different sizes was investigated by Abbasi et al. [9], and Leung et al. [10], Kim et al. [11], and Dirar et al. [12] studied the effect of CFRP reinforcements relating to the span-to-depth ratio of RC structures. In addition, the effect of steel stirrups on CFRP-reinforced RC structures was considered by Mofidi and Chaallal [13], Chen et al. [14], and de Freitas Arcine et al. [15]. Research on how mechanical parameters of the CFRP, such as fiber ratio, fiber materials, fiber layer numbers, and thickness, are related to the shear strengthening of RC structures was conducted by Jin et al. [16], Ebadi-Jamkhaneh [17], Bousselham and Chaallal [18], and Deniaud and Cheng [19]. The effects of pattern parameters, such as the space and width of CFRP strips, were studied numerically and experimentally by Tanarslan and Altin [20], Mofidi and Chaallal [21], and Khalifa and Nanni [22]. Additionally, the relationship between the angle of the CFRP strips and the behavior of the RC beams was experimentally investigated by Norris et al. [23], Chaallal et al. [24], and Monti and Liotta [25]. In addition, the effect of CFRP on the flexural strength of RC structures was studied by Vrettos et al. [26], Sun et al. [27], Smith et al. [28], and Li et al. [29]. Furthermore, research on the anchors of CFRP-strengthened structures was performed by Zhou et al. [30], Zhang and Smith [31], Sun et al. [32], Mostafa and Razaqpur [33], Pudleiner et al. [34], Li et al. [35], and Tatar et al. [36]. In addition, research on the CFRP debonding caused by various environmental conditions has recently been conducted by Zhang et al. [37], Al-lami et al. [38], and Yin et al. [39].

Most of the above studies evaluated the effect of unidirectional (vertical) CFRP strips. Compared to this, the studies on the use of bidirectional CFRP have been limited. Research papers have provided evidence of superior performances of bidirectional CFRP compared to those of unidirectional CFRP [4,40,41,42,43,44,45,46,47,48,49]. Kim et al. [40] experimentally compared the shear strengthening performance of unidirectional CFRP and bidirectional CFRP applied to I-girders. In this study, the increase in shear capacity achieved by bidirectional CFRP (up to 40%) is significantly higher than that achieved by unidirectional CFRP. A compression test of RC panels strengthened by bidirectional CFRP strips was also performed by Kim et al. [41,44] to investigate the bottle-shaped compression strut mechanism. The mechanism is easily seen in the web elements of deep RC beams. The test revealed that both the cracking loads and maximum loads of the panel were increased significantly with bidirectional CFRP. Later, Alotaibi [42] tested eight sets of RC T-beams strengthened with anchored bidirectional CFRP strips to examine the behavior. The research project presented the effectiveness of bidirectional CFRP strips related to the shear span–depth ratio of RC structures. These observations were the first attempts at investigating bidirectional CFRP. Furthermore, Haroon et al. [45] applied bidirectional CFRP strips to rectangular RC beams. The study experimentally investigated how the configuration of CFRP strips and stirrups affects the structural performance of RC beams. In addition, several studies investigated bidirectional CFRP strengthening. Rasheed et al. discussed the layout of bidirectional CFRP strips for T-beams [46]. Kalfat et al. studied the anchor system to prevent debonding [47]. Yilmaz et al. and Al-Atta et al. investigated bidirectional CFRP for slabs [48,49]. However, the studies on bidirectional CFRP strengthening are still relatively insufficient. Furthermore, they have studied the performance of bidirectional CFRP based on experiments. To define the mechanism that provides improved performance, the numerical study of bidirectional CFRP with proper mechanical models is required. Moreover, these studies have provided a few tests where the studied parameters of bidirectional CFRP strips are limited. Parameters such as the layout of horizontal CFRP and the thickness of the horizontal CFRP strips must be studied for the effective strengthening of RC structures.

The main objective of this study is to simulate the performance of RC beams strengthened by bidirectional CFRP based on numerical models. A set of RC beams with different patterns of CFRP is tested experimentally. The tested beams are numerically modeled based on finite element analysis. The numerical model is validated by comparing the result with the experiments. The numerical tests, based on the validated numerical model, are conducted to examine the parameters of the horizontal CFRP strips on RC beams. Parameters studied in this study include the layouts of horizontal CFRP strips and the number of horizontal CFRP strips.

## 2. Experimental Program

### 2.1. Code Provisions (ACI 318, 440.2R)

Since the number of experimental studies regarding bidirectional CFRP is limited, this study designed an experimental program to define the mechanism of bidirectional CFRP strips for RC beams and to verify the numerical model. To check the validity of the experiment, the design shear strengths of the test specimen were calculated and compared with the test results. In general, the shear strength of RC beams can be estimated using ACI 318-19 [50]. In general, the nominal shear strength of the RC beam, $V_{n}$, is the summation of shear strength provided by concrete ($V_{c}$) and shear reinforcement ($V_{s}$). In the code, the shear strength provided by concrete is calculated as follows:

(1) $$V_{C}=\{\begin{array}{c} \begin{array}{cc} Either\;of\{\begin{array}{c} \left[0.17\lambda \sqrt{f_{c}^{\prime }}+\frac{N_{u}}{6A_{g}}\right]b_{w}d,\;\;\;\;\;\;\;\;\;\;\;\;\;\;\;\;\;\\ \left[0.66\lambda_{s}\lambda {\left(\rho_{w}\right)}^{1/3}\sqrt{f_{c}^{\prime }}+\frac{N_{u}}{6A_{g}}\right]b_{w}d, \end{array} & for\;A_{v}\geq A_{v,min} \end{array}\\ \begin{array}{cc} \;\;\;\;\;\;\;\;\;\left[0.66\lambda_{s}\lambda {\left(\rho_{w}\right)}^{1/3}\sqrt{f_{c}^{\prime }}+\frac{N_{u}}{6A_{g}}\right]b_{w}d, & \;\;\;\;\;\;\;\;\;\;\;\;for\;A_{v}<A_{v,min} \end{array} \end{array}$$

where $b_{w}$ and d indicate the width and reinforcement depth of the rectangular section, respectively. $f_{c}^{\prime }$ is the compressive strength of the concrete. $A_{v}$ is the area of shear reinforcement within spacing s in the RC beams. $A_{v,min}$ is the minimum area of shear reinforcement within spacing s for the design. $\rho_{w}$ presents the ratio of $A_{s}$, the area of longitudinal section reinforcement, to the area of the section $b_{w}d$. $A_{g}$ is the gross area of the concrete section, and $N_{u}$ is the axial force which is normal to the cross-section, occurring simultaneously with shear force. $N_{u}$ is positive for compression and negative for tension. λ and $\lambda_{s}$ are the modification factors for the lightweight concrete and the size effect, respectively. Note that the maximum of both λ and $\lambda_{s}$ is 1. Compared to the previous version of ACI 318 (ACI 318-14) [51], the equations of $V_{c}$ have been simplified, since the previous code provided different equations depending on the axial loads.

The shear strength provided by shear reinforcement, which was not changed compared to the previous version, is defined in terms of $A_{v}$, $f_{yt}$, d, s, and α, the angle between stirrups and the longitudinal axis.

(2) $$V_{s}=\frac{A_{v}f_{yt}\left(\sin\alpha +\cos\alpha \right)d}{s}$$

To estimate the shear strength of the RC beams strengthened by CFRP, not only $V_{c}$ and $V_{s}$ but also the shear strength provided by the CFRP must be defined. ACI 440.2R-17 [52] provides formulas to calculate the contribution of the CFRP in relation to the shear strength of the structure. When the RC structure is strengthened with CFRP, the nominal shear strength of the structure can be redefined as follows:

(3) $$V_{n}=V_{c}+V_{s}+\psi_{f}V_{f}$$

$V_{f}$ is the shear strength provided by the CFRP, and $\psi_{f}$ is the reduction factor which is applied to the contribution of the CFRP system. A $\psi_{f}$ of 0.85 is recommended for a three-sided U-wrap or two-opposite-sides strengthening schemes, while a $\psi_{f}$ of 0.95 is recommended for completely wrapped members. The reduction factor for completely wrapped members is higher since the scheme is less bond dependent. Similar to $V_{s}$, $V_{f}$ is defined as follows:

(4) $$V_{f}=\frac{A_{fv}f_{fe}\left(\sin\alpha_{f}+\cos\alpha_{f}\right)d}{s_{f}}$$

where $\alpha_{f}$ is the angle between CFRP laminates and longitudinal direction. $A_{fv}$ is the cross-sectional area of the CFRP laminates with spacing $s_{f}$. For the CFRP of rectangular sections, $A_{fv}$ is the function of the width ($w_{f}$), thickness ($t_{f}$), and number ($n_{f}$) of the CFRP laminates ($A_{f}=2n_{f}t_{f}w_{f}$). $f_{fe}$ is the effective stress in the CFRP at section failure. $f_{fe}$ is directly proportional to the strain, which can be developed in the CFRP laminates at nominal strength.

(5) $$f_{fe}=E_{f}\varepsilon_{fe}$$

where $E_{f}$ is the tensile modulus of elasticity of CFRP, and $\varepsilon_{fe}$ is the failure strain of the CFRP. For RC beams completely wrapped by CFRP, $\varepsilon_{fe}$ is 0.004 and should be lower than 75% of the CFRP’s rupture failure strain, $\varepsilon_{fu}$($\leq 0.75\varepsilon_{fu}$). If CFRP systems do not wrap the entire section, $\varepsilon_{fe}$ is defined using the bond reduction coefficient $\kappa_{v}$.

(6) $$\varepsilon_{fu}=\kappa_{v}\varepsilon_{fu}\leq 0.004$$

The bond reduction coefficient is defined in terms of the concrete strength, the wrapping scheme, and the stiffness of the laminate.

### 2.2. Specimen Detail

**Figure 1:**
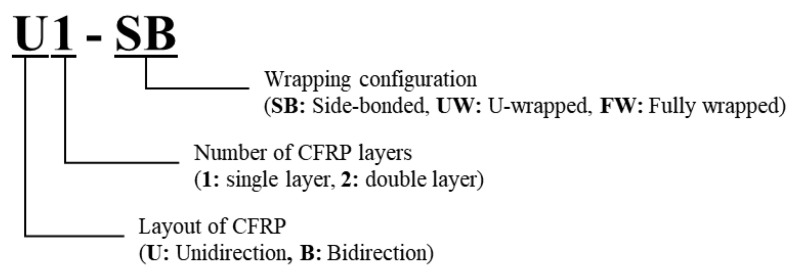
Nomenclature of test specimens.

The study focused on numerical modeling which can describe the mechanism of bidirectional CFRP strips. The experiment tested the specimens with bidirectional CFRP layouts under different CFRP configurations. Additionally, the experiment included specimens with unidirectional CFRP strips under the same configurations. The specimens used in this study were the same as the test specimens used by Haroon et al. [39]. The main variables of this study included CFRP layouts, number of CFRP layers, and wrapping configurations of CFRP strips. Figure 1 describes the nomenclature of the specimen in the experiment program. The first character represents the layout of the CFRP strip. The second character indicates the number of CFRP layers used, which was either 1 or 2. The third character describes the wrapping configuration of the CFRP.

Table 1 shows the details of the specimen. Tested RC beams had a cross-sectional dimension of 300 × 500 mm and a length of 2200 mm. The shear-span-to-depth ratio (*a*/*d*) of the specimen was 2.1. The compressive strength of the concrete ($f_{c}^{\prime }$) measured from the cylindrical concrete specimen on the 28th day was 31.8 MPa. The elastic modulus of the concrete was 27,000 MPa. The width and spacing of the CFRP strips were 100 mm and 200 mm, respectively. The same width and spacing were applied to both vertical CFRP strips and horizontal CFRP strips. The mechanical properties of the CFRP strips were tested according to ASTM D 3039. The tensile strength and elastic modulus of CFRP strips from the test were 4600 MPa and 288,900 MPa, respectively.

**Figure 2:**
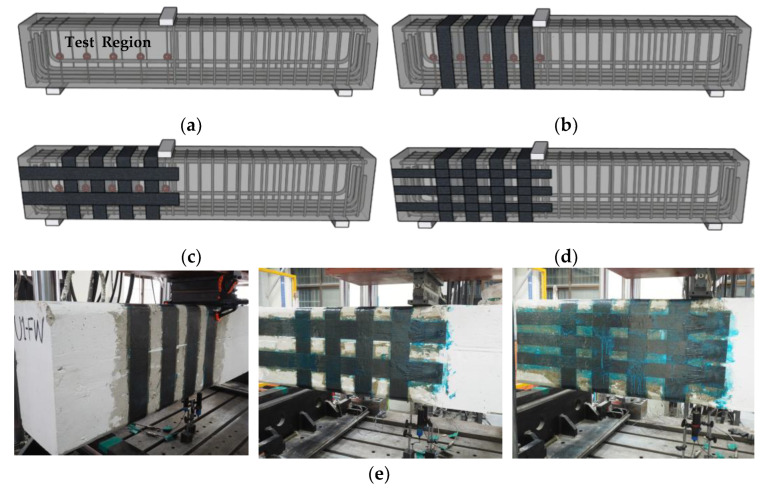
Test specimens and CFRP layout: (**a**) test region; (**b**) unidirectional CFRP layout; (**c**) bidirectional CFRP layout; (**d**) bidirectional CFRP layout (B1-FW(2)); (**e**) experiment specimens.

Figure 2 shows the specimen test region, CFRP layout, and experiment lab image. Since the test focused on shear failure, the flexural capacity of the beams was designed to be higher than the shear capacity of the beams by adding sufficient longitudinal reinforcement. D25 reinforcing bars with a yield strength of 400 MPa and an elastic modulus of 195,000 MPa were used for flexural reinforcement. The loading point was located 1020 mm from the left end of the beam, which was not symmetric. The left shear span, from the loading point to the left support, was designed to be the test region. The left span was designed to show shear failure prior to the right span. The shear reinforcement of the left span was designed using D6 rebars with 200 mm spacing, while the reinforcement of the right span was designed using D13 rebars with 100 mm spacing. B1-FW (2) had a bidirectional CFRP layout of three horizontal lines, while other bidirectional layouts had two horizontal lines of strips.

### 2.3. Test Equipment and Test Setup

**Figure 3:**
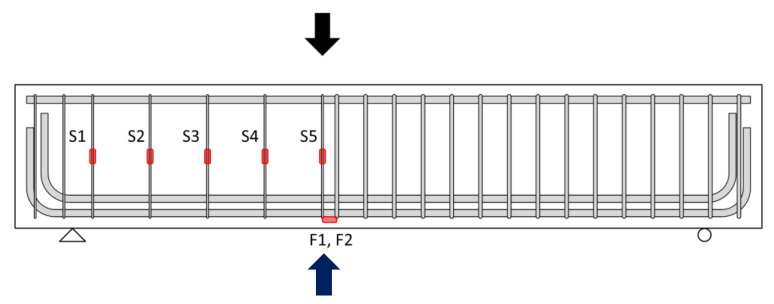
Test measurement (LVDTs and strain gauges).

The shear tests of specimens were conducted using a universal testing machine (UTM, AceoneTech, Incheon, Republic of Korea). The specimens were placed on two simple supports, and a steel plate was located on the loading point of the specimen described above. Figure 3 shows the location of the linear voltage displacement transducers (LVDTs) and strain gauge for the test. LVDTs were installed below the loading point to observe the deflection of the specimens. Five strain gauges were attached to the stirrups in the test region to measure the strain of transverse reinforcement. The strain gauges for CFRP strain measurement were attached in the same position as the strain gauge attached to the stirrups in the test region. Additionally, two strain gauges were attached to the longitudinal reinforcement at the loading point. The loading of the test specimens was achieved under a monotonic loading protocol. The specimens were unloaded when the beams reached 85% of the maximum load in the post-peak loading range. The loading was controlled to increase the deflection at a rate of 0.01 mm/sec.

### 2.4. Test Results

This section presents the test results of the CFRP-strengthened specimens. This study focused on the development of a numerical model which can describe the shear behavior of specimens. The results of the test were used for the validation of numerical models. This study summarizes the test results, and the details of the test results can be found in the previous study [45].

Table 2 shows the test results, including measured maximum strains of stirrups and strips and maximum loadings. Maximum loadings of the specimens were compared with the design capacity described in Section 2.1. The stirrups of all specimens reached the yield strain prior to the failure occurring. The test results showed higher shear capacity compared to the design shear capacity defined in ACI 318 codes. The shear ratios (*V*_test_/*V*_ACI_) were between 1.43 and 2.01. ACI 318-19 estimates the shear capacity of structures conservatively, which is reasonable.

**Figure 4:**
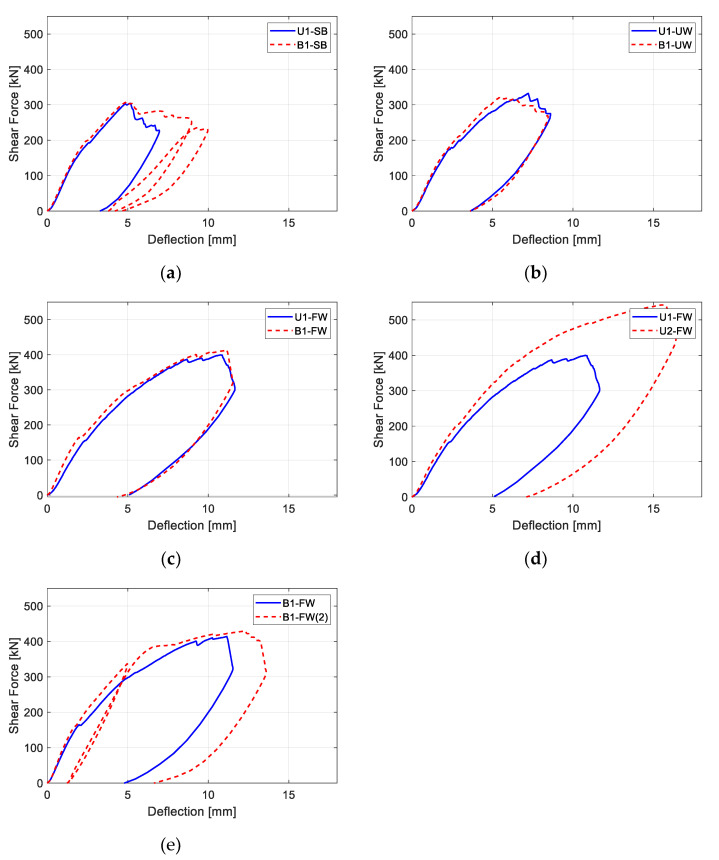
Load deflection behavior of specimens: (**a**) U1-SB vs. B1-SB; (**b**) U1-UW vs. B1-UW; (**c**) U1-FW vs. B1-FQ; (**d**) U1-FW vs. U2-FW; (**e**) B1-FW vs. B1-FW(2).

Figure 4 presents the load deflection behavior of the specimens. For the figure, shear force (y-axis) was calculated from the applied point load. In the test, the maximum shear forces of bidirectionally strengthened specimens did not show significant differences compared to those of unidirectionally strengthened specimens. However, the maximum deflections of bidirectionally strengthened specimens, with the maximum value on the x-axis, were larger than those of unidirectionally strengthen specimens. The maximum strains of the vertical CFRP strips shown in Table 2 were also higher when the bidirectional CFRP layout was applied. This reveals that the addition of horizontal CFRP strips can increase the ductility of a structure, which is preferable structural behavior.

**Figure 5:**
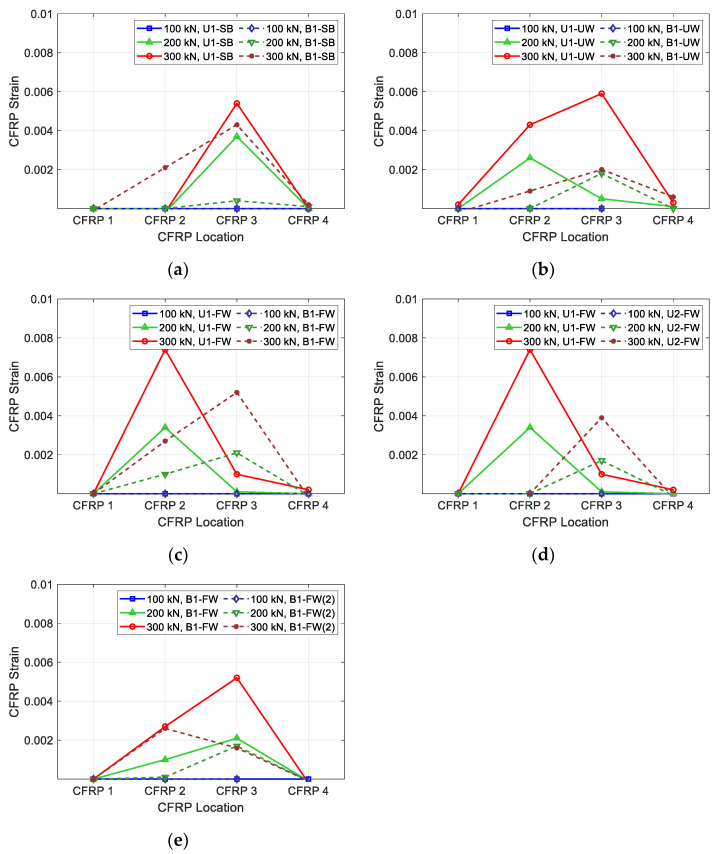
Measured CFRP strip strains of specimens: (**a**) U1-SB vs. B1-SB; (**b**) U1-UW vs. B1-UW; (**c**) U1-FW vs. B1-FQ; (**d**) U1-FW vs. U2-FW; (**e**) B1-FW vs. B1-FW(2).

Figure 5 presents the measured CFRP strains of the test specimens according to the stirrup location. CFRP 1 is the strain of the CFRP located near the left reaction point of the specimen, while CFRP 4 is the strain of the CFRP located near the loading point of the specimen. Lines with different colors represent the strains at different loading levels. At a loading level of 300 kN, the measured strain of all specimens, except U2-FW and B1-FW(2), reached the yield strain. The results indicate that the layout and configuration of CFRP strips affected the performance of the specimens. The maximum CFRP strains at a fixed load level were smaller in all specimens with bidirectional CFRP layouts than in specimens with unidirectional CFRP layouts. Additionally, side-bonded specimens showed lower maximum strains than fully wrapped and U-wrapped specimens. This is due to the failure modes of the specimens. The CFRP strips of the side-bonded specimens were delaminated before the CFRP strips reached maximum strain. For the complete analysis of the test, not only the CFRP strains but also the stirrup strains must be analyzed. The results and discussion regarding stirrup strains are provided by Haroon et al. [39].

## 3. Finite Element Analysis

**Figure 6:**
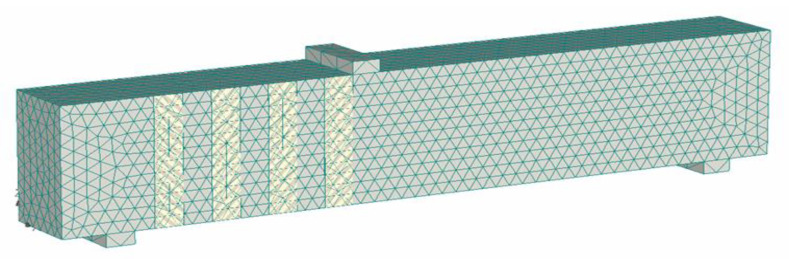
FEM modeling using ATENA 3D.

The objective of this study was to numerically investigate the parameters of CFRP strengthening, which were not discussed in the previous study [45]. The numerical model for the CFRP-reinforced concrete beams was validated by comparing the model to the test results of the previous study. To construct the numerical model for the CFRP-reinforced concrete beams, the program ATENA 3D (Cervenka, Praha, Czech Republic) [53] was utilized. ATENA 3D is nonlinear finite element analysis software that is widely used in the numerical study of concrete structures [54,55,56,57,58]. In this study, the CFRP-strengthened RC beams were modeled using the brick element (concrete) and shell element (CFRP strips). Figure 6 presents the modeling of the specimen. The gray element represents the concrete with reinforcements, and the orange element represents the CFRP strips.

For the modeling of concrete, brick and tetra meshes with the size of 0.1 m were used. CFRP strips were modeled using brick and tetra meshes with a mesh size of 0.1 m. The isotropic linear elastic material model was applied to the CFRP modeling. Between the CFRP strips and the concrete, a 3D interface describing the properties of epoxy was modeled. The model was constructed based on the study of Kalfat and Al-Mahaidi [59].

### 3.1. Validation of the Analysis Model

**Figure 7:**
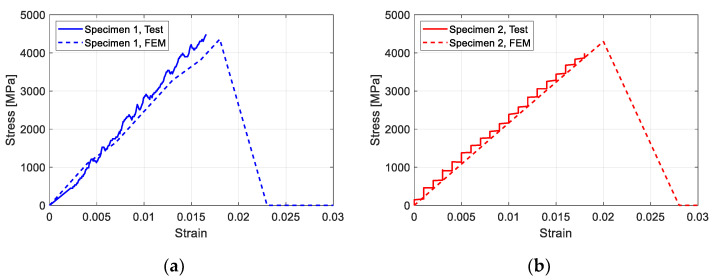
Test results of CFRP material: (**a**) CFRP specimen 1 (25 mm wide); (**b**) CFRP specimen 2 (50 mm wide).

To validate the analysis model, the results of material tests and specimens from the previous study [39] were compared with the FEM model. Figure 7 shows the CFRP material test results of the FEM. In the figure, it can be seen that the FEM model showed similar load displacement behavior to the test result. Compared to the test result, the displacement of the FEM model still increased, even with a loading smaller than the maximum loading. This was caused by the displacement control mode used in the FEM. The FEM model would have shown a similar result to the test if the analysis had been performed based on the load control.

**Figure 8:**
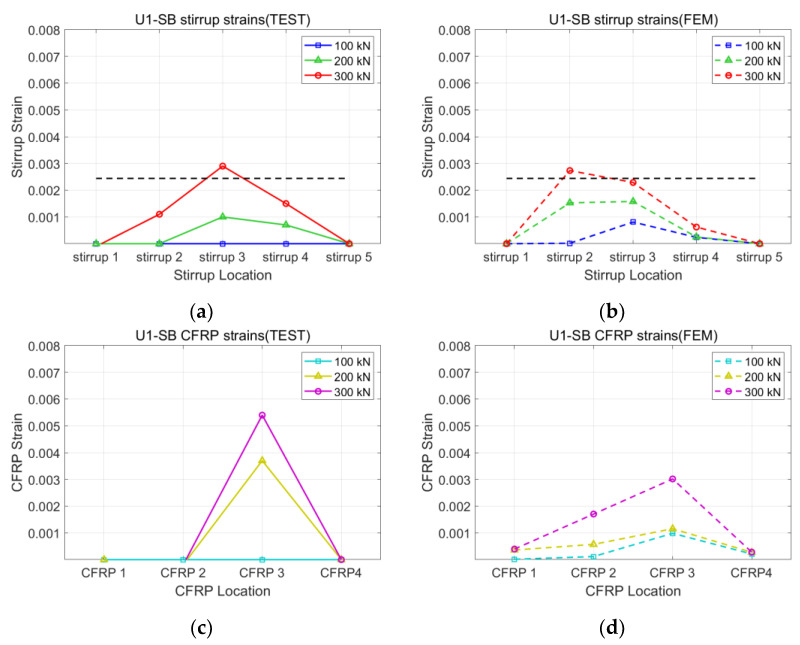
Strains of U1-SB specimen: (**a**) stirrup of test; (**b**) stirrup of FEM; (**c**) CFRP of test; (**d**) CFRP of FEM.

**Figure 9:**
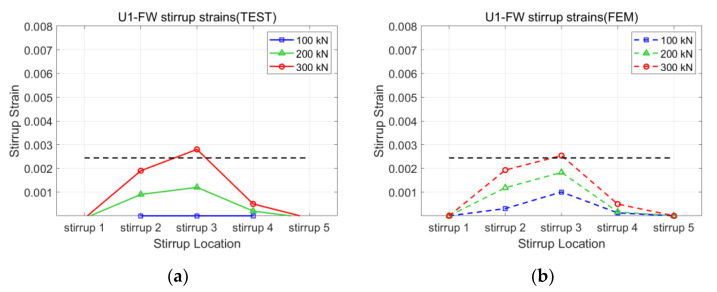

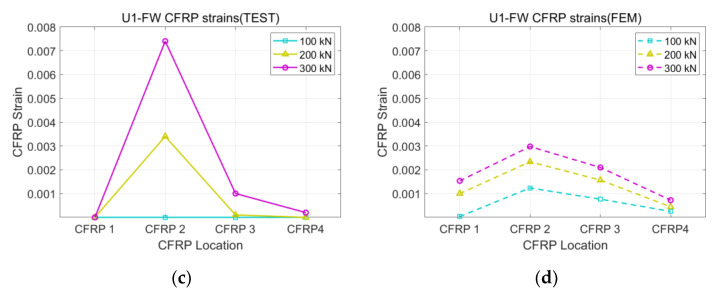
Strains of U1-FW specimen: (**a**) stirrup of test; (**b**) stirrup of FEM; (**c**) CFRP of test; (**d**) CFRP of FEM.

**Figure 10:**
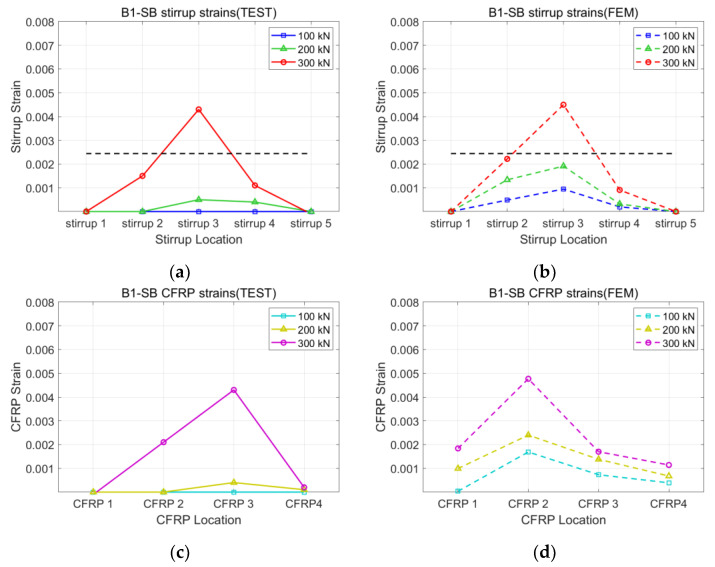
Strains of B1-SB specimen: (**a**) stirrup of test; (**b**) stirrup of FEM; (**c**) CFRP of test; (**d**) CFRP of FEM.

**Figure 11:**
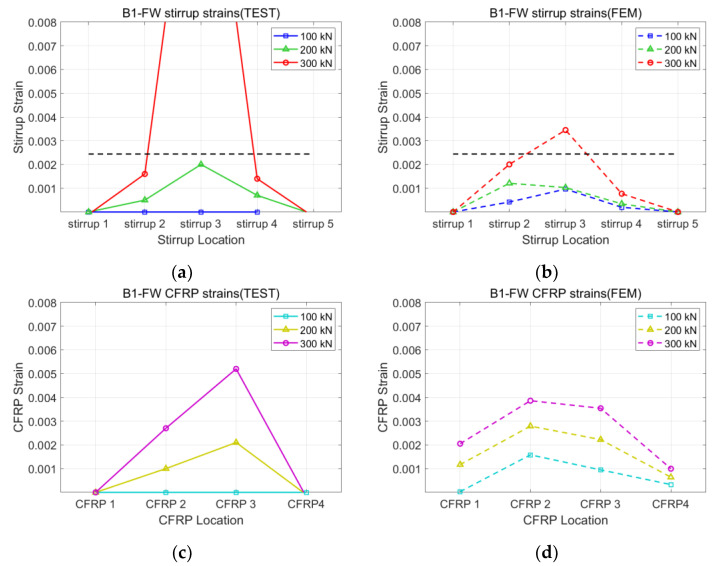
Strains of B1-FW specimen: (**a**) stirrup of test; (**b**) stirrup of FEM; (**c**) CFRP of test; (**d**) CFRP of FEM.

Figure 8, Figure 9, Figure 10 and Figure 11 present the strains of the stirrups and CFRP strips of the U1-SB, U1-FW, B1-SB, and B1-FW specimens at the different loading stages, respectively. The black, horizontal dash line represents the yielding strain of the stirrups. In Figure 8, it can be seen that the distribution of strains at different specimen locations in the FEM model was different from that in the experiment. However, the maximum stirrup strains at each loading stage were similar for both the FEM model and the experiment (0.0027 vs. 0.0029 at 300 kN). In Figure 9, the distribution of both stirrups and CFRP strains at different specimen locations in the FEM model was similar to that in the experiment. The fully wrapped CFRP configuration provides a more stable performance than the side-bonded configuration because the configuration provides a larger area of contact between the CFRP and the concrete beam. Furthermore, the fully wrapped configuration provides higher CFRP strains due to the continuing effect provided by the CFRP strips. Figure 10 and Figure 11, representing the results of the bidirectional CFRP layouts, show higher stirrup strains (0.0045 and 0.0039 at 300 kN) and lower CFRP strains (0.0043 and 0.0052 at 300 kN) compared to those in the unidirectional CFRP layouts. The overall results represent that the FEM model described the performance of the CFRP-strengthened beams well.

### 3.2. Parametric Study

**Figure 12:**
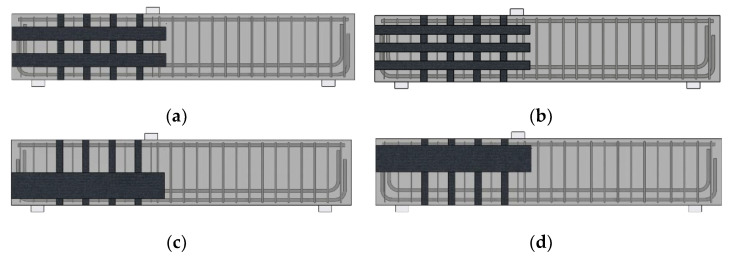
Horizontal CFRP layout patterns: (**a**) distributed CFRP layout with two strips (100 mm); (**b**) distributed CFRP layout with three strips (70 mm); (**c**) on strip (200 mm) at bottom half; (**d**) one strip (200 mm) at the upper half.

**Figure 13:**
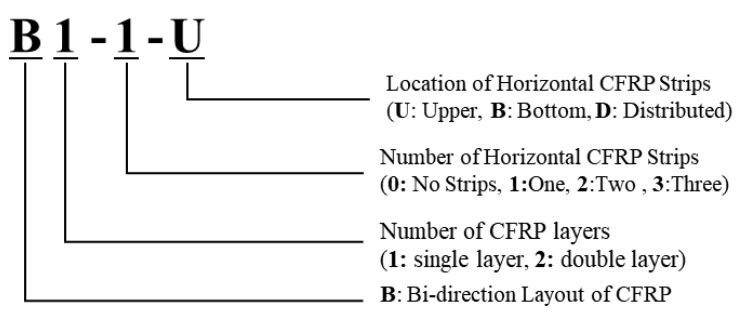
Nomenclature of test specimens for parametric study.

As mentioned, the effect of CFRP layouts on RC beams could be simulated by utilizing the FEM software. Specifically, the effect of adding horizontal CFRP strips, which was not considered in the experimental study, was examined based on the numerical model. To evaluate the effect, parameters relating to the vertical CFRP strips, such as the number of layers and the wrapping configurations, were kept consistent. The parameters of the horizontal CFRP strips, such as layout and the amounts of materials, were numerically investigated in this study. Eight numerical models with different horizontal CFRP layouts and numbers of horizontal CFRP strips were constructed. All models used the same concrete beam used in the previous section. To clearly distinguish the effect of horizontal CFRP parameters, vertical CFRP strips with 70 mm width were fully wrapped to each RC beam model. All horizontal strips were modeled as fully bonded with both ends fixed to the RC beam. Figure 12 illustrates the horizontal CFRP layout patterns used in this study. For each model, the width of a single strip and the wrapping locations were modified, while the sums of the horizontal strip width for all models were the same. Figure 12a shows when two horizontal CFRP strips of 100 mm width were used. Figure 12b shows when three horizontal CFRP strips of 70 mm width were applied. Figure 12c,d show when a horizontal CFRP strip of 200 mm width was used. One strip was attached at the upper/bottom half of the RC beam. Single strip patterns were selected to investigate whether a wide single strip could strengthen the beam effectively. In practice, not all the beams could be reinforced, as shown in Figure 12a,b, due to site conditions. Therefore, Figure 12c,d represent the limited site conditions. If these patterns had shown the strengthening performance of the RC beam effectively, it would have been helpful for the site engineers. Figure 13 presents the nomenclature of the specimens in the parametric study, and Table 3 categorizes the details of the CFRP layout considered in this section. Strains of concrete and stirrups were compared to investigate the parameters of the horizontal CFRP strips.

Table 4 presents the results of the test, including maximum strains of stirrups and strips and maximum loadings. Similar to Table 2, the maximum loadings of the models were compared with the design capacity described in Section 2.1. The *V*_test_ of the control was measured from the experiment, while the other models were measured from the FEM. For *ε*_f,max_, the values in the bracket are the strain values for the horizontal CFRP strips. As seen in the table, the stirrup strains of all models were larger than the yield strain when the failure occurred. All the test results showed a higher shear capacity compared to the designed shear capacity defined. In terms of the shear ratios (*V*_test_/*V*_ACI_), all models with bidirectional CFRP strips showed higher values than the values of the control. The shear ratios of the models were between 1.70 to 1.85. In Section 3.2.1 and Section 3.2.2, the results of the test are further discussed.

#### 3.2.1. Horizontal CFRP Layout

**Figure 14:**
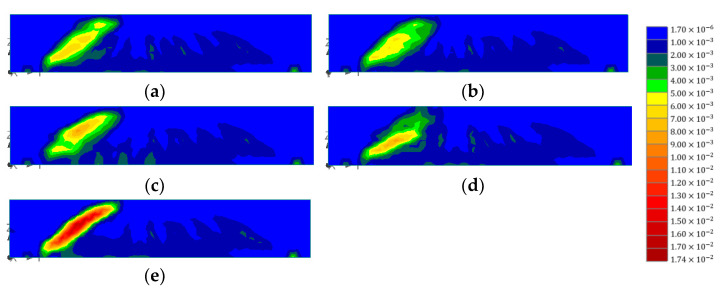
Comparison of concrete strains for different horizontal CFRP layouts: (**a**) B1-2-D; (**b**) B1-3-D; (**c**) B1-1-B; (**d**) B1-1-U; (**e**) control.

Figure 14 presents the distribution of concrete strains for the test specimens of different layouts. Control in the figure indicates the numerical model of the concrete beam strengthened with only vertical CFRP strips (no horizontal CFRP). In the figure, the high strain values of all specimens are concentrated around the diagonal crack between the left support and the left loading point. As expected, the control showed the highest strain values (0.0173) compared to the other cases. For B1-1-B and B1-1-U, the highest strains, of 0.009, were found at the upper half and the lower half of the beams, respectively. These were where the horizontal strips were not placed. Among the models with distributed horizontal CFRP strips, B1-3-D showed the lowest strain, 0.006, and the most evenly distributed results. The next lowest strain value was seen in B1-2-D (0.007). In the model, high strains were found between horizontal strips. These results indicate that the continuing effect of horizontal CFRP strips can be maximized when the strips are divided and placed widely along the depth of the concrete beam. If a wide strip is horizontally bonded aside due to the site condition, the other part without the horizontal strip is the weak point. For the effective strengthening of RC beams, horizontal strips must be bonded uniformly along the depth of the beam.

**Figure 15:**
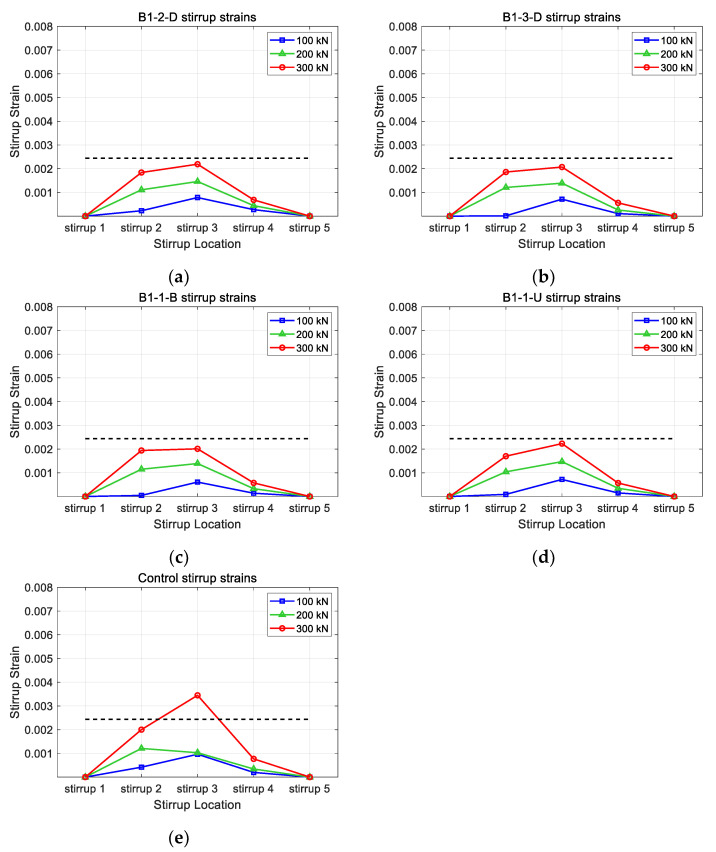
Comparison of stirrup strains for different horizontal CFRP layouts: (**a**) B1-2-D; (**b**) B1-3-D; (**c**) B1-1-B; (**d**) B1-1-U; (**e**) control.

Figure 15 presents the stirrup strains for different CFRP layouts. The distribution of stirrup strains was examined to verify the continuing effect caused by the horizontal CFRP strengthening. The maximum strain value for the B1-2-D, B1-3D, B1-1-B, B1-1-U, and control models were 0.00219, 0.00207, 0.00201, 0.00223, and 0.00345, respectively. From the figure, it can be seen that models with horizontal CFRP presented similar stirrup strain distributions regardless of layout (highest strain at the top and higher stirrup 2 strain than stirrup 4 strain at 300 kN). Stirrup strains of all models with horizontal CFRP showed lower strain values than those of the control at all loads. Furthermore, the stirrups of models with horizontal CFRP did not yield when the loading reached 300 kN, while the stirrup of the control showed a strain value higher than the yielding strain (0.00244).

#### 3.2.2. Amount of Horizontal CFRP Layout

**Figure 16:**
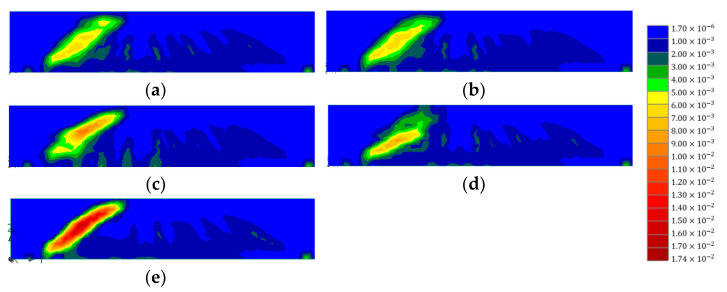
Comparison of concrete strains for different horizontal CFRP layouts: (**a**) B2-2-D; (**b**) B2-3-D; (**c**) B2-1-B; (**d**) B2-1-U; (**e**) control.

**Figure 17:**
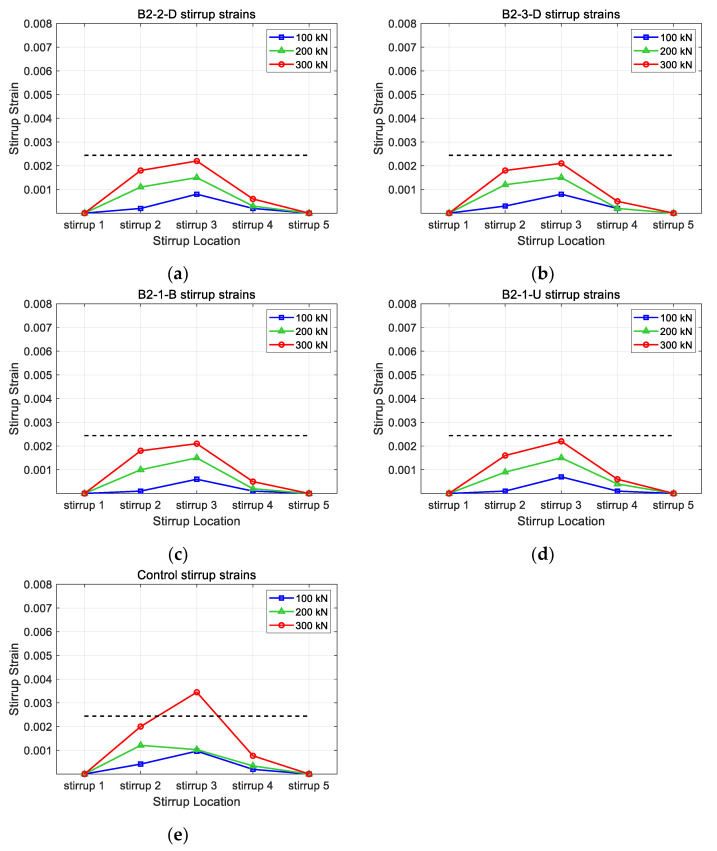
Comparison of stirrup strains for different horizontal CFRP layouts: (**a**) B2-2-D; (**b**) B2-3-D; (**c**) B2-1-B; (**d**) B2-1-U; (**e**) control.

Figure 16 and Figure 17 illustrate the concrete strain and stirrup strains with horizontal CFRP strips of 0.6 mm. As mentioned above, the vertical CFRP strips were fixed at 0.3 mm, and each thickness of the horizontal strips was set as 0.3 mm and 0.6 mm. In Figure 16, it can be seen that the patterns of concrete strains with different CFRP layouts showed similar patterns to those illustrated in Figure 14. The concentration of strains decreased as the CFRP strips were added to the concrete. Not only the strain concentration patterns but also the maximum strain values were similar for horizontal CFRP layouts. Among the models with horizontal strips, the B2-3-D model showed the lowest strain value. The maximum strain values of the B2-3-D model were similar to those of the B1-3-D model. This indicates that the increase in thickness in horizontal CFRP strips did not improve the shear performance of specimens. Although the number of horizontal strips increased, the failure came from either the delamination of the vertical CFRP or the concrete. The increase in thickness of the horizontal CFRP strips could not delay the delamination of CFRP strips. In Figure 17, it can be seen that the maximum strain value for B2-2-D, B2-3D, B2-1-B, and B2-1-U was 0.00218, 0.00214, 0.00212, and 0.00224, respectively. Figure 17 shows similar behavior. Except for the control model, stirrups did not reach the yield stress. The distribution patterns of stirrup strains were similar to those in Figure 15. Since the failure of the model came from the delamination, these are acceptable results.

## 4. Discussion

The results indicate that the layout of the horizontal CFRP affects the structural performance of RC beams significantly, while the number of horizontal CFRP strips does not significantly affect the structural performance. When a horizontal strip is located either at the bottom/upper half of the RC beam, the strips only confine the concrete and vertical strips in the located half. The horizontal strips cannot confine the other half where the high concrete strains are found. When the horizontal strip is divided into multiple strips and bonded uniformly along the depth, the effect of adding horizontal CFRP strips increases. Among the investigated models, the layout pattern with three strips provided the best performance. The pattern with three strips kept the vertical CFRP strips from debonding well compared to the others. Furthermore, the widely distributed horizontal CFRP strips confined the concrete beam and prevented the failure of the beam effectively.

Compared to the layout pattern, the number of horizontal CFRP strips cannot change the effect of horizontal CFRP strengthening. Even if the number of horizontal CFRP strip layers is doubled, the concrete strains and stirrup strains of the concrete do not decrease dramatically. The increased horizontal CFRP strip layers cannot delay the delamination of vertical CFRP strips. Although the increased horizontal strip amounts can confine the concrete for horizontal expansion, the conditions for vertical strip delamination are the same. The horizontal CFRP layout pattern becomes a more important parameter than the number of horizontal CFRP strips for the efficient strengthening of the RC beam. For the site project, the use of horizontal strips of a single layer around the depth of the beam instead of using a multiple-layered strip to a narrow location of the depth is recommended.

## 5. Conclusions

In this study, the effectiveness of bidirectional CFRP strengthening of rectangular RC beams was numerically investigated. The RC beams with CFRP strengthening were numerically modeled based on FEM. The constructed numerical models were validated by comparing them to the experiments. RC beams with unidirectional/bidirectional CFRP layout and side-bonded/fully wrapped vertical CFRP strips were used for the validation. Both the experiment and numerical model revealed that bidirectional CFRP provides improved performance compared to unidirectional CFRP. Based on the numerical model, the parameters such as the layout and amount of horizontal CFRP were studied. The layout of the horizontal CFRP is a more significant parameter than the amount of horizontal CFRP. From this study, the numerical model, which provided the performance of RC beams with bidirectional CFRP, was developed. The model can be used not only for parametric studies but also for the simulation of projects regarding CFRP strengthening of RC beams.

The following items are planned for future study. First, further study on the parameters relating to horizontal CFRP strips will be performed. Since the number of horizontal CFRP strips in this study did not show different performance, items such as the minimum number of horizontal CFRP strips for various RC beams will be studied. Next, a refinement of the numerical model will be developed. The development of a numerical model describing the behavior of CFRP-strengthened RC beams more accurately will be performed. Finally, the effect of bidirectional CFRP strengthening relating to dynamic loads will be investigated. The performance of CFRP-strengthened structures not only in relation to static loads but also to dynamic loads must be studied.

## Figures and Tables

**Table 1:** Experimental program specimen details.

Specimen	Concrete			CFRP Strip				
	b (mm)	h (mm)	$f_{c}^{\prime }$ (MPa)	$w_{f,v}\;(\mathrm{mm})$	b (mm)	h (mm)	$f_{c}^{\prime }$ (MPa)	$w_{f,v}\;(\mathrm{mm})$
U1-SB	300	500	31.8	100	200	N/A	N/A	4600
U1-UW	300	500	31.8	100	200	N/A	N/A	4600
U1-FW	300	500	31.8	100	200	N/A	N/A	4600
U2-FW	300	500	31.8	100	200	N/A	N/A	4600
B1-SB	300	500	31.8	100	200	100	200	4600
B1-UW	300	500	31.8	100	200	100	200	4600
B1-FW	300	500	31.8	100	200	100	200	4600
B1-FW(2)	300	500	31.8	100	200	100	200	4600

Note: b: width of RC beam, h: height of RC beam, $w_{f,v}$: vertical CFRP strip width, $s_{f,v}$: vertical CFRP strip spacing, $w_{f,h}$: horizontal CFRP strip width, $s_{f,h}$: horizontal CFRP strip spacing.

**Table 2:** Test results.

	*V*_ACI_ (kN)	*V*_test_ (kN)	*V*_test_/*V*_ACI_	*ε*_s,max_	*ε*_f,max_
U1-SB	212	304	1.43	0.0046	0.0062
U1-UW	213	332	1.56	0.0033	0.0073
U1-FW	219	400	1.83	0.0040	0.0170
U2-FW	269	542	2.01	0.0031	0.0103
B1-SB	212	310	1.46	0.0075	0.0047
B1-UW	213	321	1.50	0.0094	0.0024
B1-FW	219	414	1.89	0.0142	0.0103
B1-FW(2)	219	429	1.96	0.0113	0.0095

**Table 3:** CFRP strip parameters of numerical models.

Model		B1-2-D	B1-3-D	B1-1-B	B1-1-U	B2-2-D	B2-3-D	B2-1-B	B2-1-U
**Horizontal** **CFRP**	Total width (#×width) (mm)	200 (2 × 100)	210 (3 × 70)	200 (1 × 200)	200 (1 × 200)	200 (2 × 100)	210 (3 × 70)	200 (1 × 200)	200 (1 × 200)
	Thickness (mm)	0.3	0.3	0.3	0.3	0.6	0.6	0.6	0.6
**Vertical** **CFRP**	Total width (mm)	70, fully wrapped							
	Thickness (mm)	0.3							

**Table 4:** Test results for parametric study.

Model	*V*_ACI_ (kN)	*V*_test_ (kN)	*V*_test_/*V*_ACI_	*ε*_s,max_	*ε*_f,max_
Control	219	307	1.40	0.0166	
B1-2-D	219	406	1.85	0.0093	0.0136(0.0124)
B1-3-D	219	398	1.82	0.0078	0.0110(0.0098)
B1-1-B	219	376	1.72	0.0150	0.0149(0.0121)
B1-1-U	219	397	1.81	0.0190	0.0160(0.0100)
B2-2-D	219	406	1.85	0.0089	0.0130(0.0129)
B2-3-D	219	405	1.85	0.0101	0.0130(0.0117)
B2-1-B	219	372	1.70	0.0129	0.0136(0.0133)
B2-1-U	219	399	1.82	0.0185	0.0157(0.0101)

(*V*_test_ for control is from the experimental study, while *V*_test_ for the other models is from the FEM ).

## Data Availability

All the research data used in this manuscript will be available whenever requested.
